# Effectiveness of personalised risk information and taster sessions to increase the uptake of smoking cessation services (Start2quit): a randomised controlled trial

**DOI:** 10.1016/S0140-6736(16)32379-0

**Published:** 2017-02-25

**Authors:** Hazel Gilbert, Stephen Sutton, Richard Morris, Irene Petersen, Simon Galton, Qi Wu, Steve Parrott, Irwin Nazareth

**Affiliations:** a Research Department of Primary Care and Population Health, UCL, London, UK; b Institute of Public Health, University of Cambridge, Cambridge, UK; c Smokefree Camden (Public Health), NHS Camden, London, UK; d Department of Health Sciences, University of York, York, UK

## Abstract

**Background:**

National Health Service Stop Smoking Services (SSSs) offer help to smokers motivated to quit; however, attendance rates are low and recent figures show a downward trend. We aimed to assess the effectiveness of a two-component personalised intervention on attendance at SSSs.

**Methods:**

We did this randomised controlled trial in 18 SSSs in England. Current smokers (aged ≥16 years) were identified from medical records in 99 general practices and invited to participate by their general practitioner. Individuals who gave consent, were motivated to quit, and had not attended the SSS within the past 12 months, were randomly assigned (3:2), via computer-generated randomisation with permuted blocks (block size of five), to receive either an individually tailored risk letter and invitation to attend a no-commitment introductory session run by the local SSS (intervention group) or a standard generic letter advertising the local SSS (control group). Randomisation was stratified by sex. Masking of participants to receipt of a personal letter and invitation to a taster session was not possible. The personal letter was generated by a research assistant, but the remainder of the research team were masked to group allocation. General practitioners, practice staff, and SSS advisers were unaware of their patients' allocation. The primary outcome was attendance at the first session of an SSS course within 6 months from randomisation. We did analysis by intention to treat. This trial is registered with Current Controlled Trials, number ISRCTN 76561916.

**Findings:**

Recruitment, collection of baseline data, delivery of the intervention, and follow up of participants took place between Jan 31, 2011, and July 12, 2014. We randomly assigned 4384 smokers to the intervention group (n=2636) or the control group (n=1748); 4383 participants comprised the intention-to-treat population. Attendance at the first session of an SSS course was significantly higher in the intervention group than in the control group (458 [17·4%] *vs* 158 [9·0%] participants; unadjusted odds ratio 2·12 [95% CI 1·75–2·57]; p<0·0001).

**Interpretation:**

Delivery of personalised risk information alongside an invitation to an introductory session more than doubled the odds of attending the SSS compared with a standard generic invitation to contact the service. This result suggests that a more proactive approach, combined with an opportunity to experience local services, can reduce patient barriers to receiving treatment and has high potential to increase uptake.

**Funding:**

National Institutes of Health Research Health Technology Assessment.

## Introduction

Smoking is the leading cause of preventable ill health and premature mortality, accounting for more than 5 million deaths annually worldwide^1^ and 80 000 deaths in England.^2^ Although the prevalence of smoking in the adult population in Great Britain has fallen by more than half since 1974, the fall has slowed and changed little since 2007.^3^ The total direct cost to the National Health Service (NHS) of treating smoking-related disease was estimated at £5·2 billion in 2005–06 (£6·1 billion by 2016 prices).^4^

Government-funded specialist smoking cessation services, now known as NHS Stop Smoking Services (SSSs), were established by primary care trusts throughout England in 2000,^5^ to help and support smokers to quit. These services are effective,^6^, ^7^ with quit rates of around 35% at 4 weeks.^8^ This quit rate is higher than if the smokers attending SSSs had received only a prescription for a stop smoking medication.^8^ However, despite the increased probability of success, fewer than 5% of smokers attend the SSS each year and, since 2012, figures have shown a continuing downward trend.^9^, ^10^ Anecdotal evidence suggests that the increasing use of e-cigarettes as a stop smoking aid could account for this trend.^10^

Although general practitioners (GPs) and other health practitioners are encouraged to offer brief advice and to refer smokers to these services, as few as 8% of individuals report being referred.^11^ Moreover, smokers are generally expected to follow-up their referral and contact the service themselves to make the appointment,^12^ and a wide range of factors can deter smokers from seeking help. Direct marketing approaches involving proactive and personalised invitations to use the services are acceptable to smokers and could result in an increase in uptake.^13^, ^14^, ^15^ A UK study^16^ reported a 7·7% absolute increase in smokers attending the SSS when a proactive method of recruitment was used to attract smokers into the services.

**Research in context**

**Evidence before this study**

Few published studies have investigated novel methods of referral to a stop smoking service (SSS), and the present study was initiated in response to a National Institutes of Health Research Health Technology Assessment call to quantify the effect of new interventions on the numbers of smokers using National Health Service (NHS) smoking cessation services. In planning this trial, we identified studies suggesting that the direct marketing approach has potential as a population-based strategy for recruitment of smokers into support services, and that interpersonal strategies have a positive effect on recruitment into smoking cessation programmes. However, only two previous studies used proactive methods of recruitment and referral to inform smokers about such services. Lichtenstein and Hollis' study done in the USA combined a proactive approach with an introductory session, and the study by Murray and colleagues was the first in the UK to assess a proactive method of recruitment to attract smokers into the SSS. We built on these two studies by use of evidence from the Cochrane reviews that individually tailored self-help materials have a small, but useful, effect over generic materials on smoking cessation, and that the addition of personalised risk communication that is more personally relevant to the consumer has been found to increase uptake of screening.

Since the start of our study, research has been published showing that proactive offering of the services of telephone quitlines can result in an increase in uptake, and a systematic review of recruitment methods for smoking cessation programmes suggested that personal tailored messages and proactive and intensive recruitment strategies can enhance recruitment. More recently, a published study showing that a more proactive approach of Ask-Advise-Connect, whereby smokers are contacted proactively by the service upon receipt of their contact information, can reduce patient barriers to receiving treatment, and also has high potential to increase uptake.

**Added value of this study**

We extended the work of Murray and colleagues by providing a more intensive intervention, using computer-tailored feedback to deliver personalised risk information to invite and encourage people to attend the NHS SSS. To our knowledge, no other study has assessed the effects of this combined intervention in smokers. Our results add to the evidence of Lichtenstein and Hollis and of Murray and colleagues, in a larger trial, showing that personalised risk information alongside an invitation to an introductory session can increase the use of stop smoking services and lead to increased quit rates.

**Implications of all the available evidence**

SSSs offer smokers a substantially higher chance of stopping smoking than does attempting to quit without support. The evidence suggests that a programme of proactive recruitment can be effective in raising awareness of the SSS, and personal invitations offer an opportunity to promote the services in the form of introductory sessions to emphasise its approachability and empathy. Further research is needed to distinguish the effects of the two components of the intervention.

Smokers underestimate their own personal risk of illness compared with that of other smokers;^17^ thus, a key aim in motivating smokers to make a quit attempt is to persuade them that these risks are personally relevant. Computer tailoring uses information about individual characteristics to personalise and tailor communications,^18^ and individually tailored self-help materials can have a small but useful effect over generic materials for smoking cessation.^19^ Tailored smoking cessation advice can also include personalised risk communication based on an individual's own risk factors, which is more relevant than information about population average risks.^20^ Individual risk information can also arouse fear or concern, which might prompt a quit attempt, particularly when combined with a reassuring message that adoption of the recommended action would be effective.^21^, ^22^ The Health Belief Model emphasises the importance of provision of a specific cue to action, which can act as a trigger and increase adoption of the recommended behaviour.^23^ This combination of hard-hitting “why quit” messages about the consequences of tobacco use, and supportive and positive “how to quit” messages, emphasising quitting resources, has been shown to be effective in some mass media campaigns.^24^, ^25^ Additionally, smokers might be deterred from seeking help because of low awareness or inadequate information about available services.^26^ Offering a no-commitment introductory session as a way to experience the service can build awareness of, and comfort with, the quitting services.^24^ This approach, combined with personalised communications to patients, has the potential to engage with a larger proportion of the smoking population.^15^

We did the Start2quit trial to assess the effectiveness of a two-component personalised intervention on attendance at the SSS, and on biochemically validated 7 day point-prevalent abstinence.

## Methods

### Study design and participants

We did this randomised controlled trial in 18 SSS areas in England, with 99 general practices within the SSS areas. The trial was originally funded for 48 months, with plans to recruit practices from ten SSSs. Additional funding was approved in July, 2012, 2 years after the initial funding of the trial by the National Institute for Health Research (NIHR), to increase the sample size to allow sufficient power to assess the main secondary outcome of 7 day point-prevalent abstinence. The study did not otherwise deviate from the original protocol.

The NIHR England Primary Care Research Network recruited SSSs and practices. We targeted areas of high deprivation with large ethnic minority populations, in which smoking prevalence is high. Current smokers were identified from medical records in participating practices. GPs screened these records to exclude any severely or terminally ill individuals. Those remaining on the list were sent an invitation letter from their GP to participate, together with a participant information sheet, a consent form, and a screening questionnaire. Only one participant per household was invited to participate. Eligible individuals were aged 16 years or older, could read English, were motivated to quit, and had not attended the SSS in the previous 12 months. Motivation to quit was defined as an individual answering “yes” to either or both of two questions: are you seriously thinking of quitting in the next 6 months? Would you think of quitting if appropriate help were offered at a convenient time and place? The screening questionnaire assessed eligibility, demographics, self-reported health, nicotine dependence, smoking history, determination and confidence to quit. We sought consent to use information from medical records and from the screening questionnaire to generate personal messages about quitting, and for researchers to access data for SSS attendance. Non-responders were sent a reminder and duplicate questionnaire after 3 weeks. Eligible smokers who returned the questionnaire and signed the consent form were randomly assigned to the intervention or control groups. Patients had the opportunity to decline participation, but to return the questionnaire with basic information to update their smoking status in their medical records.

The protocol was approved by the South West London Research Ethics Committee.

### Randomisation and masking

Participants were randomly assigned (3:2) within practice, via computer-generated permuted block randomisation (block size of five), to receive either an individually tailored risk letter and invitation to attend a no-commitment “come and try” introductory session run by the local SSS or a standard generic letter advertising the local SSS. Randomisation was stratified by sex. The use of a computer program that implemented randomisation after consent and baseline data entry ensured that allocation was concealed and selection bias eliminated. The same computer program combined the data from the baseline questionnaire and from patient medical records with the correct messages from a message library to generate tailored letters and invitations to the taster session for participants assigned to the intervention group, and the generic letters for those assigned to the control group.

Masking of participants to receipt of a personal letter and invitation to a taster session was not possible. Although the personal letter was generated in the practice by a research assistant, the remainder of the research team were masked to group allocation. In follow-up interviews, the interviewer was masked to the allocation of the respondent to avoid bias in outcome assessment. Interviewers could become unmasked during the course of the interview by information volunteered by the participants; however, the main outcome questions were asked at the start of the interview. GPs, practice staff, and SSS advisers were not aware of their patients' allocation. Randomisation at the level of participant rather than by practice meant there was a slight risk of contamination by communication between patients at the same practice allocated to different intervention groups. This risk was reduced by only one person from the same household being invited to participate.

### Procedures

Participants allocated to the control group were sent a standard generic letter from the GP practice, which advertised the local SSS and asked the smoker to contact the service to make an appointment to see an adviser. Participants allocated to the intervention group received a brief personalised and tailored letter sent from the GP that included information specific to the patient, obtained via the screening questionnaire and from their medical records; a personal invitation and appointment to attend a “come and try it” taster session to find out more about the services, run by advisers from the local SSS; and a repeated personal letter with a further invitation 3 months after the original for participants who did not attend a taster session after the first letter and invitation.

The aims of the letter were to communicate personal risk level of serious illness if the individual continued to smoke, by use of personalised information, and to encourage attendance at the SSS. The letter was accompanied by a personal invitation to a taster session held roughly 2 weeks after the invitation was mailed, with details of time and place. The recipient did not have to book an appointment, just to turn up at the specified time. The goals of the taster session were to provide information, to promote the SSS, to address any concerns or queries participants might have had, and to encourage sign up to a course. This session was not intended to replicate the first session of an SSS course. The appendix provides a full description of the intervention.

At the end of the 6 month follow-up period in each SSS, attendance data were collected from the SSSs using NHS monitoring data. Additionally, a computer-assisted telephone interview was done 6 months after the date of randomisation by research interviewers, independent from the service providers, to assess self-reported SSS attendance, current smoking status, and other outcome data. If no response was received or if the participant was unable to complete the telephone interview, a paper version of the follow-up questionnaire was sent by post. If a participant did not wish to complete the telephone interview or paper questionnaire, the interviewer attempted to ask four basic questions relevant to the primary and main secondary outcome. The number of participants attending the taster sessions was derived from records of attendance. Participants claiming abstinence at follow-up were asked to provide a salivary cotinine sample by post, by use of a saliva sample kit,^27^ to biochemically validate 7 day point-prevalent abstinence,^28^ with a cotinine cutoff concentration of 12 ng/mL.^29^ Use of nicotine replacement therapy and e-cigarettes at the time of the sample was assessed by questionnaire. Returned saliva samples were packaged in dry ice and posted to Salimetrics (Newmarket, Suffolk, UK)—an independent laboratory specialising in analysis of biological samples. The appendix shows details of the timing of assessments, intervention, and follow-up.

### Outcomes

The primary outcome measure was attendance at the first session of a 6 week SSS course, over a period of 6 months from the receipt of the invitation letter, measured by records of attendance at the SSSs. The main secondary outcome measure was 7 day point-prevalent abstinence at 6 months' follow-up, validated by salivary cotinine. Other secondary outcome measures were additional periods of abstinence (24 h and 7 day point-prevalent, 1 month and 3 month prolonged) measured by self-report, and validated 3 month prolonged abstinence; self-reported quit attempts and changes from baseline in daily cigarette consumption, and in motivation and intention to quit in continuing smokers; and the number of participants completing the 6 week SSS course.

Process measures included the number of smokers attending the taster session, and the perception of both the personal risk letter and of the taster session, based on questions included in the telephone interview at the 6 month follow-up.

Economic evaluation was done alongside the trial to estimate short-term cost-effectiveness of the two interventions from an NHS and personal social services perspective, as recommended by National Institute for Health and Care Excellence (NICE) guidance.^30^ We estimated the costs of provision of the interventions and the costs of patients' use of health and social care services. Costs were expressed in pounds sterling (£) at a 2012–13 price base. The primary outcome for the economic evaluation was assessed in terms of quality-adjusted life-years (QALYs) based on patients' responses to the EuroQol five dimensions (EQ-5D) health-related quality-of-life questionnaires.^31^ We did an additional model-based analysis to extrapolate the expected lifetime cost-effectiveness of the intervention compared with control.

Anonymised data comprising sex, date of birth (converted to age at the time of the invitation), and home postcode (converted to an Index of Multiple Deprivation [IMD] score—the government's official measure of multiple deprivation at small area level^32^) of patients invited to participate in the study, but who declined or did not respond, were collected to establish the external validity of our results.

### Statistical analysis

The statistical analysis plan is available online. On the basis of evidence from a study by Murray and colleagues,^16^ we conservatively estimated an increase in SSS attendance of 4·6% (from 8·9% to 13·5%; odds ratio [OR] 1·65), requiring 1029 participants per group (N=2058) to detect a statistically significant difference at the 5% level with 90% power. We originally planned to recruit practices from ten SSSs. The taster sessions in each SSS were to be run by the same four advisers, comprising ten therapist clusters. Thus, before adjustment for clustering, we would expect 103 patients per cluster. We assumed a therapist intraclass correlation coefficient (ICC) of 0·005, requiring further inflation of the sample size by a factor of 1·51 in the intervention group in which the effects would occur. Thus, 1554 participants would receive the tailored letter and invitation to the taster session (N=2583), equivalent to a randomisation ratio of 3:2.

The study by Murray and colleagues^16^ also reported validated 6 month quit rates of 4% in the intervention group versus 2·2% in the control group (a difference of 1·8%). Our planned sample size of 2583 participants provided less than 80% power to detect a difference of 1·8%; however, if the intervention were to cause the quit rate to double from 2·2% to 4·4% (difference of 2·2%) there would have still been 80% power to detect such a difference. The trial extension to enable evaluation with adequate power of the intervention effect on 7 day point-prevalent abstinence at the 6 month follow-up required an 80% increase in the sample size, to 1793 in the control group and 2707 in the intervention group (N=4500), assuming the same therapist effect as the original protocol. This increase would give 85·4% power to detect a difference of 1·8% at the 5% significance level, assuming quit rates of 4% in the intervention group and 2·2% in the control group. The same sample size would provide 95% power to detect the difference between quit rates of 4·4% and 2·2% (doubling of quit rate).

Introduction of this particular secondary outcome, in view of its importance in assessment of the intervention, necessitated the consideration of multiple significance testing. We therefore planned to split the permitted type 1 error rate of 0·05 between the original primary outcome and this key secondary outcome.

We initially estimated that six practices, with a list size of more than 4000 patients, in each of ten SSSs would contribute about 240 000 patients and, assuming a conservative smoking prevalence of 15% in patients aged 16 years and older, 36 000 smokers. On the basis of previous studies,^16^, ^33^ we estimated a response rate of 7% from smokers motivated to quit, from two mailings to secure 2520 participants, which would meet the requirements of the original sample size calculation. The extension to the trial required an additional 2000 participants and, in view of good recruitment rates at the time the extension was funded, we estimated that an additional eight SSSs (48 practices) would recruit 2060 participants, giving a total of 4580 participants and meeting the requirement of the new power calculation.

Comparison of proportions was done for binary outcomes between the intervention and control groups, by use of logistic regression with a random intercept model to allow for possible correlation within SSSs. We deemed this univariable analysis as primary, but multivariable analysis was also done to account for potential imbalance in baseline characteristics (sex, age, IMD score, dependence score, intention to quit, determination to quit, longest previous quit attempt, living with other smokers, and previous SSS attendance). Self-reported quit attempts and changes in daily cigarette consumption and in motivation and intention to quit in continuing smokers were compared descriptively.

We assessed interactions between intervention and deprivation (defined in quintiles), intervention and sex, and intervention and age (defined by categories 16–39 years, 40–64, and >65 years), for the primary outcome and 7 day point-prevalent abstinence at the 6 month follow-up. Analysis of the interaction by social deprivation was specified in the original protocol. The analyses by sex and age were added when we prepared our analysis plan. Planned subsidiary analyses were done to examine any delayed effect of sending repeat reminders to smokers on the uptake of service. We also report post-hoc analyses of differences in recruitment in SSSs, follow-up response, and outcome between SSSs.

The prespecified statistical analysis plan, which was agreed and approved by the trial steering committee before database lock, was strictly adhered to, in keeping with International Comparison Program harmonisation. We did analysis by intention to treat. For abstinence, the standard assumption was that participants lost to follow-up were still smoking.^34^ Analysis was done with Stata (version 13). This trial is registered with Current Controlled Trials, number ISRCTN 76561916.

### Role of the funding source

The funder of the study had no role in study design, data collection, data analysis, data interpretation, or writing of the report. HG, RM, and IP had full access to all the data in the study, and HG had final responsibility for the decision to submit for publication.

## Results

Recruitment, collection of baseline data, delivery of the intervention, and follow up of participants took place between Jan 31, 2011, and July 12, 2014. We randomly assigned 4384 participants to the intervention group (n=2636) or to the control group (n=1748); 4383 participants comprised the intention-to-treat population (figure).

Table 1 shows baseline characteristics. We noted some differences between participants enrolled and individuals who declined the invitation or did not reply: there were fewer males in the participants group than in the non-participants group (2231/4383 [50·9%] *vs* 56 046/106 451 [52·7%]), participants were older than non-participants (mean age 49·3 years [SD 14·3] *vs* 43·3 years [15·9]), and the IMD score was lower in participants than in non-participants (mean score 24·3 [SD 14·2] *vs* 25·5 [14·6]), showing a slightly higher level of deprivation in non-participants.

Objective data for attendance at the SSS were obtained for all participants from SSSs at the end of the 6 month follow-up period. Additional self-report data were obtained from 3372 (77%) participants (appendix p 10). The proportion of participants completing 6 month follow-up did not differ between groups (figure). We recorded some differences in characteristics between participants who completed the follow-up and those who did not (appendix p 11).

Of 630 participants claiming abstinence at 6 months, 595 (94·4%) agreed to send a saliva sample for biochemical validation of 7 day abstinence and 443 (70·3%) returned a sample. 399 (90·0%) samples were sent for analysis (n=279 from the intervention group and n=120 from the control group); samples from 44 (10%) participants who reported that they had resumed smoking between follow-up and returning the sample were not analysed. Of the samples analysed, 345 (86·5%) were validated (243 [87·0%] of 279 from the intervention group and 102 [85·0%] of 120 from the control group]): 249 (72·1%) samples contained less than 12 ng/mL cotinine, 36 (10·4%) samples indicated use of nicotine replacement therapy, and 60 (17·3%) samples indicated use of e-cigarettes. Of the 54 (13·5%) samples not validated, 30 (55·5%) samples contained more than 12 ng/mL cotinine and indicated no use of nicotine replacement therapy or e-cigarettes, five (9·3%) participants had reported that they had smoked in the previous 6 days, and 19 (35·1%) samples were insufficient for analysis.

The proportion of people attending the first session of a 6 week SSS course during the 6 month period from receipt of the invitation was significantly higher in the intervention group than in the control group (17·4% *vs* 9·0%; unadjusted OR 2·12 [95% CI 1·75–2·57]; p<0·0001; table 2). The proportion of people completing the 6 week SSS course was likewise significantly higher in the intervention group than in the control group (14% *vs* 7%; unadjusted OR 2·24 [95% CI 1·81–2·78], p<0·0001; table 2). 7 day point-prevalent abstinence at 6 month follow-up, validated by salivary cotinine, was significantly higher in participants in the intervention group than in those in the control group (9·0% *vs* 5·6%; unadjusted OR 1·68 [95% CI 1·32–2·15], p<0·0001), as it was for all other periods of abstinence measured by self-report, and 3 month validated prolonged abstinence (table 2).

Cigarette consumption was slightly reduced in individuals who continued to smoke, and almost a quarter had made a quit attempt (table 3). Intention and motivation to quit changed little in continuing smokers, with few differences in these measures between groups (table 3).

The intervention effect was significantly greater in men than in women for attendance at the SSS and for validated 7-day point-prevalent abstinence (table 4). The intervention effect on attendance at the SSS also differed significantly by IMD quintile, although this difference was due to attendance being lower in the control group for participants in quintiles 2 and 3 rather than higher in the intervention group (table 4).

Attendance at the SSS over the 6 months from randomisation peaked after the first taster sessions, with little increase after a repeat invitation was sent (appendix p 12). Recruitment rates differed greatly between the SSSs, ranging from 2% to 6%, as did follow-up response rates (appendix p 13). Moreover, there were large variations between practices within SSSs. We deliberately included some practices in areas with high ethnic populations, but recruitment was especially low in these practices (appendix p 14). We recorded some variation in outcome between SSSs. Overall attendance between SSSs varied from 2% to 23% (ICC 0·031 [95% CI 0·01–0·09]), and validated 7 day abstinence from 2% to 13% (0·034 [0·011–0·096]), suggesting that around 3% of participants' tendency to attend and to quit smoking was explained by the SSS in which they were located.

Of the 2635 participants in the intervention group invited to attend a taster session, 739 (28%) attended. In the intervention group, more participants who had attended a taster session attended the SSS than those who did not (338 [45·7%] of 739 *vs* 120 [6·3%] of 1896), and more participants who attended a taster session and the SSS achieved validated 7 day abstinence (97 [28·7%] of 338) than those who only attended the taster session (40 [10·0%] of 401), or only attended the SSS (21 [17·5%] of 120). Participants who did not attend a taster session or the SSS had the lowest rates of validated 7 day abstinence (78 [4·4%] of 1776 in the intervention group and 74 [4·9%] of 1590 in the control group).

Of 2910 (66·4%) participants who completed the telephone follow-up, the letter was more often read by participants in the intervention group than by those in the control group (1517 [87·2%] of 1740 *vs* 977 [83·5%] of 1170) and more participants in the intervention group discussed the letter with others (654 [37·5%] *vs* 348 [29·7%]). The personal risk letter was considered to be acceptable, with most respondents rating it easy to read (1421 [95·8%] of 1484), easy to understand (1440 [96·8%] of 1488), interesting (888 [61·2%] of 1452) and useful (960 [66·0%] of 1452). Very few respondents reported feeling angry (60 [4·1%]), anxious (122 [8·2%]) or depressed (67 [4·5%]). The taster sessions were viewed positively by the majority of attendees; 334 (73·9%) of 452 found the session interesting and 329 (72·8%) of 452 found it useful.

The mean total intervention cost was £777 (SD £2176) in the intervention group and £679 (£1860) in the control group. The intervention has a 20–27% probability of being deemed more cost effective at 6 months than the control strategy on the basis of NICE decision-making thresholds. However, the probability that the intervention is the most cost-effective option was 83% when a lifetime horizon was adopted, which suggests that the intervention represents a cost-effective use of NHS resources.

## Discussion

Our findings show that an intensive intervention delivering personalised risk information together with an invitation to attend a no-commitment taster session designed to inform smokers about the service and what it offers more than doubled the odds of attending the SSS compared with a standard generic invitation to contact the service. Additionally, participants in the intervention group were more than twice as likely to complete the 6 week SSS course. These results support and extend previous evidence that a more proactive approach, combined with an opportunity to experience local services, can reduce patient barriers to receiving treatment and has high potential to increase uptake.^15^, ^16^, ^35^ We also found a significantly higher rate of validated 7 day point-prevalent abstinence at 6 months in participants in the intervention group than in those in the control group. The rate of abstinence was also higher for all other periods of abstinence at the 6 month follow-up point.

The intervention was particularly effective among men. Typically, more women than men attend and set a quit date with the services.^2^ This intervention encouraged more men than women to attend. If, by use of recruitment methods such as this, more men can be persuaded to use the support, the number of unaided and unsuccessful attempts made by men to quit smoking could be reduced.

Traditionally, SSSs target smokers with an intention to quit in the next 2 weeks, and concern has been expressed that smokers recruited proactively might be less likely to quit than would those who self-referred.^36^ Despite recruitment of a high proportion (42·5%) of participants with no immediate plans to quit, and 12·6% with no plans to quit, the overall abstinence rate in those attending the SSS was similar to estimates of longer-term abstinence in smokers setting a quit date with the SSS.^6^ Treatments do not only help during a quit attempt and help to prevent relapse, but can also increase motivation to quit.^37^ Tzelepis and colleagues^38^ also pointed out that if counselling were offered only to smokers ready to quit, a large proportion of proactively recruited smokers would miss out on getting effective support. Thus, recruitment of smokers with more distant plans to quit might result in more successful attempts and long-term abstinence than if these smokers were left to make unplanned attempts on their own.

We recorded some variation in both attendance and 7 day validated abstinence between SSSs. There is known to be wide variation in outcomes between SSSs.^39^, ^40^ Furthermore, SSSs were going through a substantial change in commissioning during the recruitment period for this study, and we do not know how this might have affected our findings. Organisational and service differences could also have a key influence on attendance following the taster session.

Both parts of the intervention had demonstrated acceptability. The taster sessions were regarded as helpful and reassuring, and built the intended awareness and comfort with the services. Very few respondents reported perceiving the letter as antagonistic or depressing, or reported feeling anxious because of the letter, suggesting that the balance of risk information with awareness of the availability of support was appropriate.^41^

This is the first study to examine the effect of use of introductory taster sessions to encourage people to attend a smoking cessation service. The strategy of proactive recruitment approach via mass mailing was a strength of the study, and enabled recruitment of a more representative sample of smokers than if more traditional reactive recruitment methods were used. The strategy also allowed us to target at-risk groups and disadvantaged people, and to deliver cessation services to poorer communities^14^—a priority of the SSS—with the result that more than half of practices were located in areas of high deprivation. A further strength was the collection of anonymised data for individuals who were invited but did not agree to take part, allowing us to assess external validity. Another strength was that the primary outcome was an objective measure of attendance, obtained for all participants.

Our study has some limitations. Although we achieved a good geographical spread, we included only 18 of the 151 SSSs in England, and these participating SSSs might not necessarily be representative of all SSSs in England. A further limitation is that, despite our proactive recruitment strategy, the recruitment rate of 4·1% of potentially eligible smokers who were sent an invitation was low and, as a result, we recruited only a small proportion of smokers in each area. However, the sample was representative of smokers in terms of sex and deprivation, but we did not manage to recruit a sufficient number of younger smokers. Attendance at the SSS tends to be concentrated in older age groups, and it is important to attract younger smokers.

In view of the research design we used for this study, we could not estimate the relative contribution of the two intervention components on attendance and abstinence. Further research in the form of a factorial study (whereby participants are randomised to receive a personalised invitation to a local SSS, or an invitation to a taster session, or both, or neither) is necessary to estimate the separate effects of the two components. Implementation studies are also needed to confirm the findings of this study and establish a real-world effect size. In implementation of this intervention to all smokers in the practice, information available in medical records could be used to generate a personal invitation to a taster session. This approach would eliminate the need for return of an assessment questionnaire and obtaining of consent, and could result in increased attendance.

Compared with the generic letter, the tailored letter plus the taster session is likely to be more effective, but also more costly. In the short term (6 months), the intervention is less likely to be cost effective compared with the control. However, smoking cessation yields long-term health-care cost savings and health benefits attributable to the reduced risk of smoking-related diseases. The long-term economic model indicates that the intervention has a great probability of being more cost effective than the control strategy over a lifetime horizon. A full cost-effectiveness analysis has been done and will be published elsewhere.

The number of smokers accessing the SSS has decreased significantly in the past few years.^10^ Efforts to reverse this trend should be a priority, because services offer smokers a substantially higher chance of stopping smoking than does attempting to quit without support. Our findings suggest that a programme in general practice of proactive recruitment using personal tailored letters and introductory sessions can more than double the odds of attending the SSS, and lead to increased quit rates.

## Figures and Tables

**Figure:**
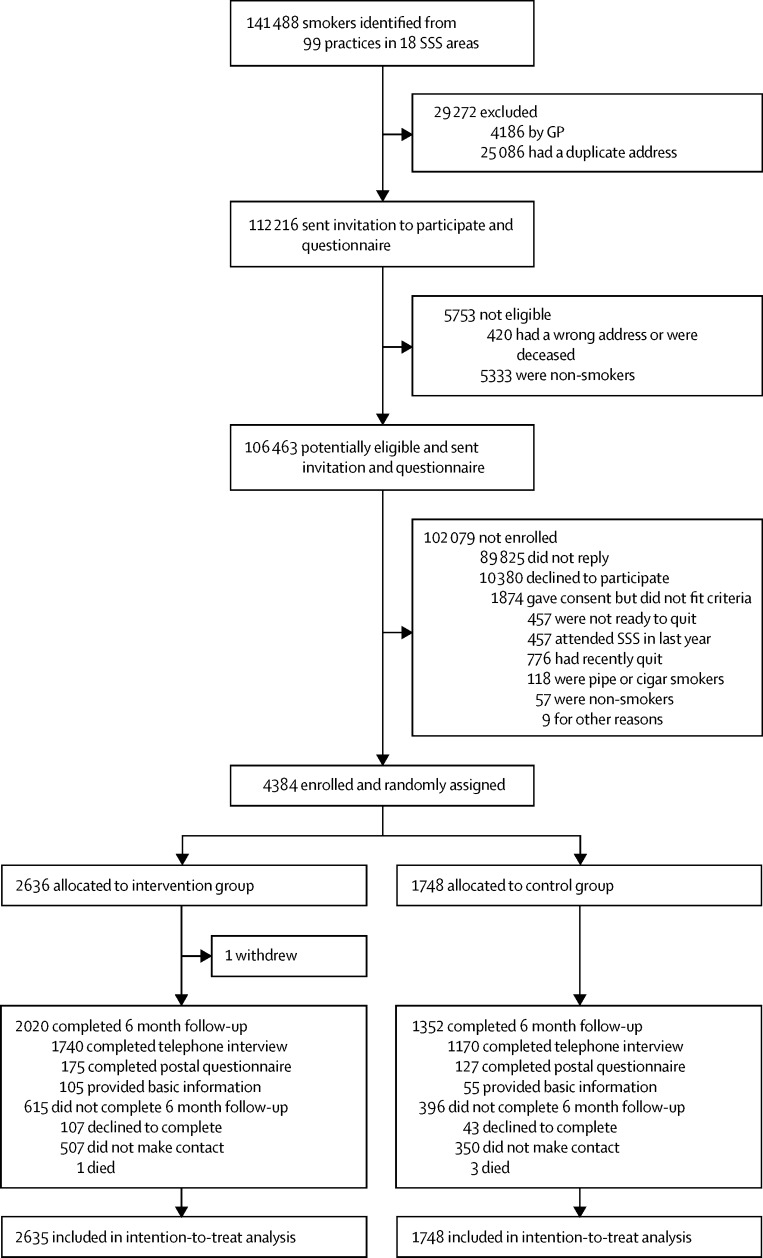
Trial profile *Total list size of 962 548 individuals (range of list sizes per practice 2205–26 000). GP=general practitioner.

**Table 1:** Baseline characteristics

		**Intervention group (n=2635)**	**Control group (n=1748)**
**Demographics**			
Sex			
	Male	1345 (51·0%)	886 (50·7%)
	Female	1290 (49·0%)	862 (49·3%)
Age (years)			
	Mean (SD)	49·2 (14·3)	49·5 (14·3)
	Median (range)	50 (16–88)	50 (16–89)
Marital status			
	Single	664 (25·2%)	444 (25·4%)
	Living with a spouse	1429 (54·2%)	961 (55·0%)
	Separated or divorced	392 (14·9%)	252 (14·4%)
	Widowed	134 (5·1%)	83 (4·7%)
Employment status			
	Unemployed	287 (10·9%)	190 (10·9%)
	Paid employment	1422 (54·0%)	903 (51·7%)
	Full-time student	44 (1·7%)	32 (1·8%)
	Home maker	104 (4·0%)	89 (5·1%)
	Retired	495 (18·8%)	344 (19·7%)
	Disabled or too ill to work	254 (9·6%)	171 (9·8%)
Highest qualification			
	None	672 (25·5%)	460 (26·3%)
	GCSE, CSE, or O Level	1042 (39·5%)	655 (37·5%)
	A Level	306 (11·6%)	232 (13·3%)
	Degree or equivalent	454 (17·2%)	301 (17·2%)
	Postgraduate	82 (3·1%)	34 (1·9%)
Ethnic origin			
	White	2522 (95·7%)	1669 (95·5%)
	Other*	113 (4·3%)	79 (4·5%)
Deprivation quintile (IMD)			
	1	334 (12·7%)	215 (12·3%)
	2	378 (14·3%)	244 (14·0%)
	3	574 (21·8%)	392 (22·4%)
	4	677 (25·7%)	453 (25·9%)
	5	653 (24·8%)	436 (24·9%)
Living with smokers		835 (31·7%)	567 (32·4%)
**Smoking characteristics**			
Daily smoker		2401 (91·1%)	1616 (92·4%)
Cigarettes per day			
	Mean (SD)	16·1 (8·6)	16·8 (9·9)
	Median (IQR)	15 (0·1–80)	15 (0·3–99)
Time from waking to first cigarette			
	<5 min	568 (21·6%)	414 (23·7%)
	6–30 min	1186 (45·0%)	802 (45·9%)
	31–60 min	436 (16·5%)	246 (14·1%)
	1–2 h	222 (8·4%)	152 (8.7%)
	>2 h	215 (8·2%)	132 (7.6%)
Dependence score (0–6)†		2·57 (1·49)	2·67 (1·52)
	Low (0–2)	1094 (41·5%)	669 (38·3%)
	Medium (3)	850 (32·3%)	581 (33·2%)
	High (4–6)	673 (25·5%)	487 (27·9%)
Age started smoking			
	Mean (SD)	16·5 (4·5)	16·5 (4·6)
	Median (range)	16 (6–55)	16 (1–51)
**Intention and motivation to quit**			
When planning to quit			
	Next 2 weeks	481 (18·3%)	315 (18·0%)
	Next 30 days	606 (23·0%)	380 (21·7%)
	Next 6 months	1103 (41·9%)	759 (43·4%)
	Not in the next 6 months	333 (12·6%)	218 (12·5%)
Longest previous quit attempt			
	<24 h	243 (9··2%)	172 (9·8%)
	1–6 days	474 (18·0%)	286 (16·4%)
	1–4 weeks	436 (16·5%)	282 (16·1%)
	>1 month	1454 (55·2%)	986 (56·4%)
Previously attended SSS		872 (33·1%)	613 (35·1%)
Mean score when asked, “How much do you want to quit?”‡		3·74 (0·91)	3·79 (0·90)
Mean score when asked, “How determined are you to quit?”‡		3·74 (0·93)	3·75 (0·93)
Mean score when asked, “How confident are you that you can quit?”‡		2·73 (1·07)	2·69 (1·06)
**Health**			
Health problems (self-reported)		592 (22·5%)	424 (24·3%)
Health problems (number of QOF diseases recorded)§			
	0	1422 (54·0%)	957 (54·7%)
	1	758 (28·8%)	459 (26·3%)
	2	313 (11·9%)	222 (12·7%)
	3	106 (4·0%)	75 (4·3%)
	4	29 (1·1%)	25 (1·4%)
	5	5 (0·2%)	7 (0·4%)
	6	1 (0·04%)	3 (0·2%)
	7	1 (0·04%)	0
Pregnant		2 (0·1%)	9 (0·5%)
On HRT		35 (1·3%)	19 (1·1%)
On the contraceptive pill		124 (4·7%)	76 (4·3%)

Data are n (%), unless otherwise specified. GCSE=General Certificate of Secondary Education. CSE=Certificate of Secondary Education. O Level=General Certificate of Educational Ordinary Level. A Level=General Certificate of Educational Advanced Level. IMD=Index of Multiple Deprivation. SSS=Stop Smoking Service. QOF=Quality and Outcomes Framework. HRT=hormone replacement therapy.

* Non-white or missing.

† Dependence score was computed from the number of cigarettes per day and time from waking to first cigarette.

‡ Score ranges from 1 (not at all) to 5 (extremely).

§ The QOF register is a system for the performance management and payment of general practitioners in the National Health Service in England, Wales, Scotland and Northern Ireland, and includes indicators for managing some of the most common chronic diseases (eg, asthma, diabetes) and major public health concerns (eg, smoking, obesity).

**Table 2:** Primary and secondary outcomes at 6 month follow-up

	**Intervention group (n=2635)**	**Control group (n=1748)**	**Unadjusted OR (95% CI)**	**p value**	Adjusted OR (95% CI)*	**p value**
Attendance at SSS	458 (17·4%)	158 (9·0%)	2·12 (1·75–2·57)	<0·0001	2·20 (1·80–2·70)	<0·0001
7 day point-prevalent abstinence (validated)	236 (9·0%)	97 (5·5%)	1·68 (1·32–2·15)	<0·0001	1·67 (1·29–2·14)	<0·0001
7 day point-prevalent abstinence (self-report)	424 (16·1%)	187 (10·7%)	1·61 (1·34–1·94)	<0·0001	1·62 (1·34–1·97)	<0·0001
24hr point-prevalent abstinence (self-report)	445 (16·9%)	201 (11·5%)	1·57 (1·31–1·88)	<0·0001	1·57 (1·31-1·89)	<0·0001
1 month prolonged abstinence (self-report)	357 (13·5%)	151 (8·6%)	1·67 (1·36-2·04)	<0·0001	1·70 (1·38–2·10)	<0·0001
3 month prolonged abstinence (self-report)	240 (9·1%)	103 (5·9%)	1·61 (1·26–2·04)	<0·0001	1·64 (1·28–2·11)	<0·0001
3 month prolonged abstinence (validated)	150 (5·7%)	60 (3·4%)	1·70 (1·25–2·31)	0·001	1·68 (1·23–2·30)	0·001
Number completing 6 week NHS course	382 (14·5%)	123 (7·0%)	2·24 (1·81–2·78)	<0·0001	2·30 (1·84–2·87)	<0·0001

Data are n (%), unless otherwise specified. OR=odds ratio. SSS=Stop Smoking Service. NHS=National Health Service.

* Adjusted for sex, age, Index of Multiple Deprivation score, dependence score, intention to quit, determination to quit, longest previous quit attempt, living with other smokers, and previous SSS attendance.

**Table 3:** Self-reported changes in daily cigarette consumption, quit attempts, and changes in motivation and intention to quit in continuing smokers

		**Intervention group (n=2190)**	**Control group (n=1547)**	**Total (N=3737)**
Change in daily cigarette consumption		−2·6 (6·4)	−2·7 (6·7)	−2·6 (6·5)
Number who made a quit attempt		528 (24·1%)	359 (23·2%)	887 (23·7%)
Changes in motivation and intention				
	Change in intention to quit (3 to −3)	−0·28 (1·11)	−0·28 (1·07)	−0·28 (1·1)
	Change in want to quit (4 to −4)	0·05 (1·11)	0·04 (1·08)	0·05 (1·08)
	Change in determination to quit (4 to −4)	−0·01 (1·24)	0·04 (1·25)	0·01 (1·24)
	Change in confidence in quitting (4 to −4)	0·10 (1·31)	−0·02 (1·22)	0·05 (1·27)

Data are mean (SD) or n (%). The subgroup of continuing smokers was not representative of all participants randomly assigned, but was defined post-baseline.

**Table 4:** Subgroup analyses of 6 month outcomes

			**Intervention group (n=2635)**	**Control group (n=1748)**	**OR (95% CI)**	**p**_interaction_
**Attendance at SSS**						
Sex			..	..	..	0·01
Male			255/1345 (19·0%)	71/886 (8·0%)	2·70 (2·04 − 3·57)	
Female			203/1290 (15·7%)	87/862 (10·1%)	1·67 (1·28 − 2·19)	
Age (years)			..	..	..	0·65
	16–40		77/664 (11·6%)	31/431 (7·2%)	1·69 (1·09 − 2·63)	
	40–64		283/1576 (18·0%)	92/1048 (8·9%)	2·28 (1·78 − 2·94)	
	≥65		98/395 (24·8%)	35/277 (12·6%)	2·33 (1·52 − 3·57)	
Interaction with deprivation						
Deprivation quintile (IMD score)			..	..	..	0·001
		1	58/334 (17·4%)	22/215 (10·2%)	1·85 (1·09 − 3·14)	
		2	70/378 (18·5%)	19/244 (7·8%)	2·65 (1·55 − 4·54)	
		3	106/574 (18·5%)	17/392 (4·3%)	5·00 (2·94 − 8·49)	
		4	113/677 (16·7%)	47/453 (10·4%)	1·76 (1·22 − 2·54)	
		5	109/653 (16·7%)	53/436 (12·2%)	1·46 (1·02 − 2·07)	
**7 day point-prevalent abstinence (validated)**						
Sex			..	..	..	0·01
	Male		131/1345 (9·7%)	39/886 (4·4%)	2·37 (1·63 − 3·42)	
	Female		105/1290 (8·1%)	58/862 (6·7%)	1·23 (0·88 − 1·72)	
Age (years)			..	..	..	0·72
	16-40		46/664 (6·9%)	21/431 (4·9%)	1·47 (0·86 − 2·50)	
	40-64		150/1576 (9·5%)	57/1048 (5·5%)	1·83 (1·33– 2·51)	
	≥65		40/395 (10·1%)	19/277 (6·9%)	1·53 (0·87 − 2·70)	
Deprivation quintile (IMD)			..	..	..	0·68
	1		34/334 (10·2%)	15/215 (7·0%)	1·51 (0·80-2·85)	
	2		44/378 (11·6%)	18/244 (7·4%)	1·63 (0·91-2·90)	
	3		60/574 (10·5%)	23/392 (5·9%)	1·92 (1·16-3·18)	
	4		58/677 (8·6%)	19/453 (4·2%)	2·14 (1·26-3·64)	
	5		39/653 (6·0%)	22/436 (5·0%)	1·18 (0·69-2·03)	

Data are n/N (%), unless otherwise specified. OR=odds ratio. IMD=Index of Multiple Deprivation.
