# Supplementary material for: Effectiveness of personalised risk information and taster sessions to increase the uptake of smoking cessation services (Start2quit): a randomised controlled trial
Source: Lancet. 2017 Feb 25;389(10071):823–33. doi: 10.1016/S0140-6736(16)32379-0 (PMC5357975; doi:10.1016/S0140-6736(16)32379-0)
Supplement: Supplementary appendix [file mmc1.pdf]

THE LANCET

Supplementary appendix

This appendix formed part of the original submission and has been peer reviewed. We post it as supplied by the authors.

Supplement to: Gilbert H, Sutton S, Morris R, et al. Effectiveness of personalised risk information and taster sessions to increase the uptake of smoking cessation services (Start2quit): a randomised controlled trial. *Lancet* 2017; published online Jan 24. http://dx.doi.org/10.1016/S0140-6736(16)32379-0.

Supplementary material

1) Development of the intervention
2) Supplementary Figure 1: Study Schedule showing the duration and timing of the procedure, intervention and follow-up
3) Supplementary Table 1. Number of participants completing the follow-up questionnaire.
4) Supplementary Table 2. Characteristics associated with study attrition
5) Supplementary Figure 2. Time from randomization to attendance at the SSS in the intervention (n=445) and control groups (n=147)
6) Supplementary Table 3. Response within SSS
7) Supplementary Tables 4 and 5. Attendance at the SSS and abstinence in SSS attenders by intention to quit

1) **Development of the intervention**

Development of the tailored intervention letter

The overall objectives of the letter were to communicate personal risk level if the person continues to smoke, using individualised information on the risk of serious illness, and to encourage attendance at the SSS.

Letter content

The letter was tailored to the individual using characteristics from practice records (gender and age) and confirmed by the baseline screening questionnaire, information obtained from the screening questionnaire (number of cigarettes per day and previous quit attempts) and information from medical records about diagnosed conditions on the Quality and Outcomes Framework (QOF) register. The final list of diseases consisted of: all cancers (excluding lung due to its terminal nature); myocardial infarction; coronary heart disease (CHD) and heart failure (combined because of terminology and the difficulty of telling someone they have a 'weak heart'); lone atrial fibrillation; stroke and transient ischaemic attack (TIA); diabetes; epilepsy; hypertension; hypothyroidism; asthma; chronic obstructive pulmonary disease (COPD); dementia (if the patient was able to consent); depression, severe mental illnesses (schizophrenia and bipolar disorders); and obesity. Possibilities around co-occurrence of diseases were considered and messages created for the following: COPD and heart failure; COPD and asthma; CHD and hypertension, stroke, dementia, severe mental illnesses, diabetes, obesity; and for multiple conditions. Because of additional risks associated with smoking for women who are pregnant, taking the contraceptive pill or hormone replacement therapy (HRT), personal risk information was also included for these smokers.

Personal risk information can be presented as an absolute or relative risk score, categorised, or as a list of the individual's risk factors. As it was felt not appropriate to provide specific probability figures without the opportunity to discuss them with a health professional, risk was classified as high, very high or extremely high compared to non or ex-smokers, as recommended by Edwards et al.[13]

The offer of help was tailored to previous quit experience, and the letter was accompanied by a personal invitation to the taster session with details of time and place.

The content of the letter was developed in collaboration with GPs and primary care experts with knowledge of medical information available in records.

Letter structure

The letter was headed 'Personal Health Risk Report and Taster Session Invitation' which appeared under the practice letter heading and logo. It consisted of four sections (see Table below). The amount of tailoring was maximised within the constraints of the short screening questionnaire and a brief letter, so that the final communication consisted of two pages. The section headings were coloured as a traffic light system, using red for the risks, orange to encourage the person to prepare to stop, and green for the invitation to the taster session.

The letter was generated by a computer program, signed by the GP and sent from the practice. The first letter was posted to the participant within three weeks of returning the completed questionnaire. A second identical letter and invitation was sent three months later to every participant who had not attended one of the earlier taster sessions.

	Objectives	Information included	Source
1) Introduction	• To explain purpose of letter, so that the individual will know that it is a personalised letter based on their assessment.		
2) Personal risk in terms of dependence and general health	• To tell the individual their dependence in the context of norms.<br>• To indicate a category of risk according to dependency in terms of the number of cigarettes smoked per day, the number of QOF registered conditions, and age.	Dependence<br>Age<br>Number of QOF diseases	Questionnaire<br>Medical records
3) Disease specific health risks and the benefits of	• To make the individual aware of the personal health consequences of continuing to smoke, and their own individual risk of serious illness in relation to dependence and own health status	Dependence<br>Age<br>Gender<br>QOF disease	Questionnaire<br>Medical records

quitting	• To make the individual anxious because of perception of their own personal risk<br>• To change the individual's balance of perceived 'benefits' against their understanding of the harm caused by smoking.		
4) Invitation to taster session	• To remind the individual that help and support is immediately available<br>• To encourage them to seek out support and use the resources available	Previous quit attempts	Questionnaire

Example of the personal letter

PRACTICE LETTERHEAD

Personal Health Risk Report and Taster Session Invitation

Dear

You recently filled in a questionnaire for the start2quit project. This letter is based on your answers in the questionnaire and your medical records. It is written for you personally and gives you advice about smoking. We are also inviting you to a Taster Session to help you to become smokefree and improve your health.

Your personal risk

Based on your smoking habits and your personal health, your current risk of developing a serious illness and suffering an early death is very high compared to a non-smoker or ex-smoker of your age.

You may think that you are not affected by smoking, but smokers are more likely to get all kinds of cancer, heart conditions and lung disease. Even by smoking 12 cigarettes per day you are seriously increasing your risk of developing one of these major diseases and dying sooner than you need to. Your records show that you are also pregnant and smoking during pregnancy also harms your baby, and can lead to complications and miscarriage.

Take control and change your life

The good news is that if you quit now, at 45, you can halve your additional risk of contracting these diseases, or of suffering any other conditions such as a stroke and osteoporosis. You will also drastically reduce the increased risks to your unborn child. By going smokefree now, even though you do not yet have any symptoms, you are more likely to live longer, and we recommend that you consider quitting without delay. It could well be the best thing that you will ever do for yourself.

Don't do it alone

You might think it is hard to stop but you don't have to do it alone. Help and support is available. The NHS Stop Smoking Service offers free personal support to help you to quit. Even though you previously have not quit for more than 24 hours, joining a stop smoking group or getting one-to-one support will increase your chances of becoming smokefree. You will also feel less alone and gain the support of other people who are quitting.

A place is reserved for you

So that you can find out more about the Stop Smoking Service, we are inviting you to a 'Come and Try it' session at Islington Town Hall on Tuesday 22nd February 2011, at 6.15pm. Please bring the Invitation Card enclosed with you. If you cannot attend this session, please contact Leanne Gardner on *****. We can offer you an alternative time or an immediate appointment with an advisor.

With very best wishes

Lead General Practitioner

Development of the taster session and training

The goal of the taster session was to offer information about the SSS, to promote the service, to address any concerns or queries smokers may have about the service provided and encourage sign up to a course. It was not intended to replicate the first session of a course.

A draft of the content of the taster sessions was prepared by one of the co-investigators (SG), and was developed into a standard protocol in consultation with the research team. The final standardized protocol also included items from the NHS Centre for Smoking Cessation and Training's Standard Treatment Programme[45] to ensure conformity with national guidelines, and a detailed manual was produced. The standard protocol for the taster session included:

- a motivational element, congratulating attendees on coming to the session
- an introduction to the SSS, emphasising that it is a free service, and based on well-researched evidence emphasising the importance of stopping smoking and outlining the benefits of quitting, both health and financial / lifestyle
- information about the services offered, outlining the structure of the treatment programme in one-to-one or group sessions, the length of sessions and of the course
- information about what to expect when they attend and the content of advice, e.g. emphasizing that no-one will be forced to quit, but will be helped to explore the reasons for and against wanting to give up smoking, and helped to develop strategies to resist smoking after their quit date
- interaction between attendees, discussing, among other things, reasons for stopping
- discussion about withdrawal symptoms, with information about the available medications and range of nicotine replacement therapy (NRT) products available
- the opportunity to have a measurement of CO level taken, with an interpretation
- a 5-min DVD showing group and one-to-one SSS sessions in progress, and testimonials from previous successful attendees, produced by UCL Media Services in collaboration with Camden SSS
- the opportunity to ask questions about the service
- details of how to contact the service and a clear and persuasive invitation to sign up for a group or individual session

Between two and seven advisors in each SSS, already trained to give smoking cessation advice in group and one-to-one sessions, attended a two hour training session to enable them to facilitate the taster sessions according to the standardised protocol and manual. The training sessions took place within each SSS and were led by two members of the team (either HG or SG), and included an explanation and clarification of the study protocol and procedures, and specified the exact information to be delivered in the session. Only trained advisors led the taster sessions, and each session was run either by one advisor with additional administrative support provided by one other, or the presentation was divided between two advisors. They were encouraged to introduce themselves and describe their background and expertise to reassure attendees of their credibility and expertise.

2) **Supplementary Figure 1: Study Schedule showing the duration and timing of the procedure, intervention and follow-up**

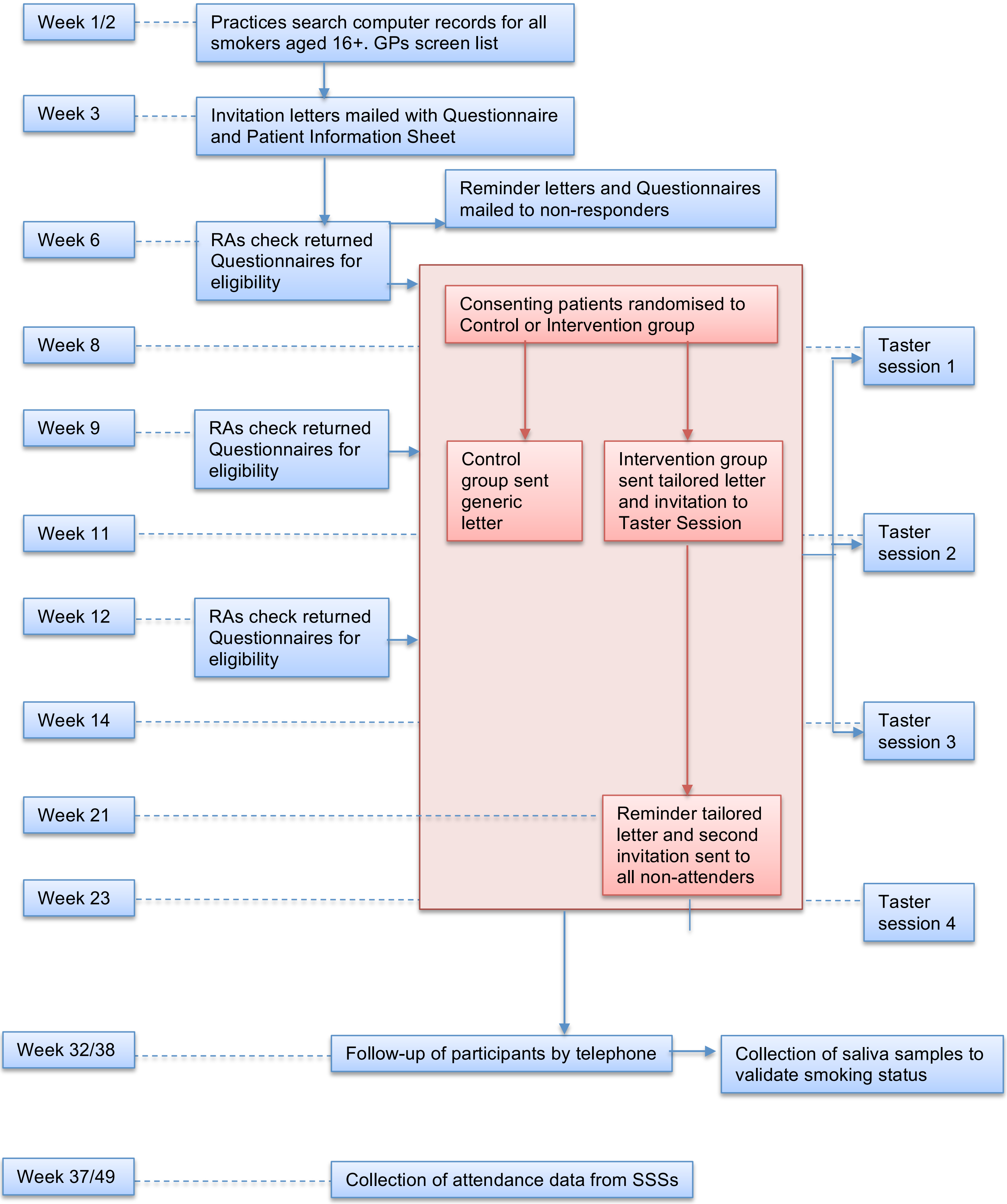

3) **Supplementary Table 1. Number of participants completing the follow-up questionnaire.**

		Intervention group	Control group	Total
Telephone interview		1740(66·0%)	1170(66·9%)	2910(66·4%)
Postal questionnaire		175(6·6%)	127(7·3%)	302(6·9%)
Basic questions		105(4·0%)	55(3·1%)	160(3·7%)
	Total	2020(76·7%)	1352(77·3%)	3372(76·9%)

4) **Supplementary Table 2. Characteristics associated with study attrition**

	Completed follow-up n(%)/mean (SD)	Not completed follow-up n(%)/mean (SD)	P value
	3354 (76·9)	1005 (23·1)	
Mean Age(SD)	50·51(13·75)	45·28(15·29)	<0·0001
Deprivation (IMD score)			
Quintile 1	459 (83·6)	90 (16·4)	<0·0001
Quintile 2	493 (79·3)	129 (20·7)	
Quintile 3	742 (76·8)	224 (23·2)	
Quintile 4	872 (77·2)	258 (22·8)	
Quintile 5	787 (72·3)	302 (27·7)	
Marital status			<0·0001
Single	774 (69·9)	334 (30·1)	
Not single	2580 (79·4)	671 (20·6)	
Mean Dependence score (0-6) (SD)	2·57 (1·49)	2·73 (1·54)	=0·004
Longest previous quit attempt			
<24 hrs			
1-6 days	292(70·4)	123(29·6)	=0·003
1-4 weeks	572(75·3)	188(24·7)	
>1 month	557(77·6)	161(22·4)	
	1911(78·3)	529(21·7)	
When planning to quit			
In next 2 weeks	573(72)	223(28)	=0·001
Next 30 days	755(76·6)	231(23·4)	
Next 6 months	414(77·8)	1448(22·2)	
Not in next 6 months	445(80·8)	106(19·2)	
Previously attended SSS			
No	2184(75·4)	714(24·6)	=0·0006
Yes	1188(80·0)	297(20·0)	
Mean score 'How much do you want to quit?'(1–5)	3·73(0·9)	3·86(0·91)	<0·0001
Mean score 'How determined are you to quit?'(1–5)	3·71(0·94)	3·85(0·93)	<0·0001
Mean score 'How confident are you that you can quit?'(1–5)	2·69(1·06)	2·79(1·09)	=0·005

5) **Supplementary Figure 2. Time from randomization to attendance at the SSS in the intervention (n=445) and control groups (n=147)**

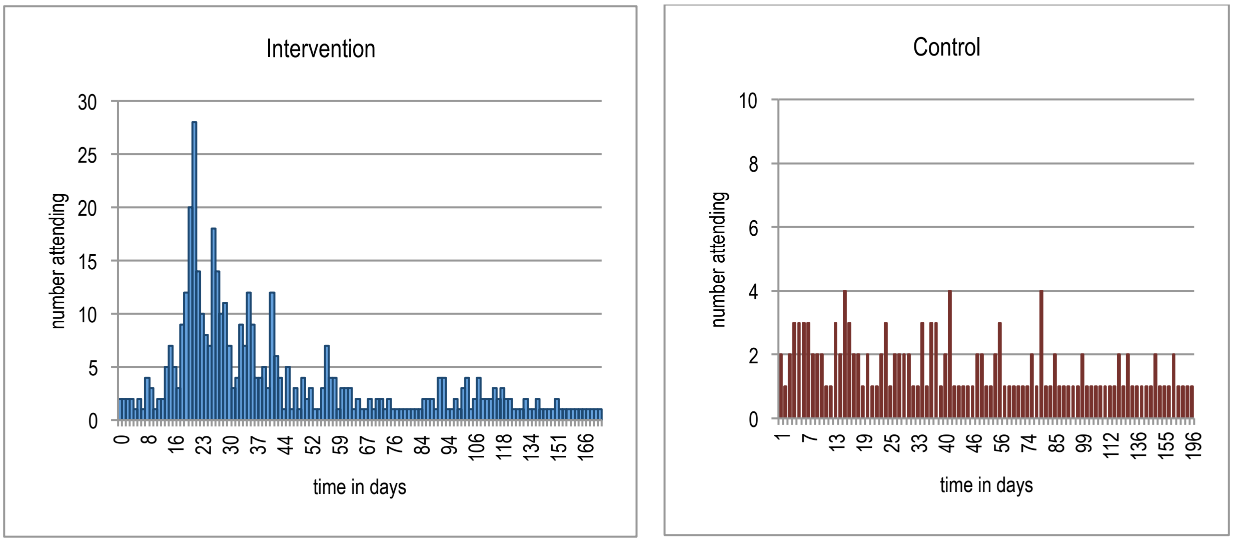

6) **Supplementary Table 3. Response within SSS**

	SSS	Recruitment rate (%)	Recruitment rate range between practices (%)	6-month follow-up response (%)	6-month follow-up response range between practices (%)
1	Camden	3·2	2·7-5·8	65·6	61·0-78·6
2	Oxfordshire	6·7	5·5-9·2	72·1	69·7-74·1
3	Medway	5·1	1·8-5·9	72·2	68·2-80·0
4	Eastern and Coastal Kent	5·5	3·5-8·2	80·4	72·3-85·7
5	Lincolnshire	3·1	2·0-3·6	82·8	82·6-83·3
6	Essex	5·0	3·0-6·1	75·3	57·1-87·0
7	Cornwall	5·3	4·3-6·5	76·9	74·6-80·0
8	Derby	3·4	1·7-4·1	67·0	60·0-72·5
9	Brent	2·3	1·4-3·9	79·2	68·4-100
10	Plymouth	3·3	1·6-5·1	70·2	64·3-75·5
11	Swindon	4·7	3·8-6·1	74·9	68·3-79·3
12	Durham and Darlington	4·0	2·3-5·7	76·5	61·5-82·6
13	Hampshire	4·6	2·7-5·6	79·6	72·3-86·7
14	Portsmouth	3·0	1·3-5·1	82·9	74·3-96·8
15	Staffordshire	3·8	2·0-6·1	81·1	75·0-84·3
16	Barnsley	3·2	2·7-4·4	76·8	72·3-79·7
17	Buckinghamshire	4·5	3·7-5·4	85·3	82·0-93·3
18	Coventry	2·7	1·9-4·9	87·0	77·8-100

7) **Supplementary Tables 4 and 5.**

Supplementary Table 4. Attendance at the SSS by intention to quit n=4195

	Intervention n=2523 n(%)	Control n=1672 n(%)	Total n(%)
When planning to quit			
next 2 weeks	92 (19·1%)	41 (13·0%)	133 (16·7%)
next 30 days	148 (24·4%)	45 (11·8%)	193 (19·6%)
next 6 months	167 (15·1%)	57 (7·5%)	224 (12·0%)
not in next 6 months	30 (9·0%)	9 (4·1%)	39 (7·1%)

Supplementary Table 5. Validated abstinence in SSS attenders by intention to quit n=589

When planning to quit	Validated abstinence	Intervention n=437 n(%)	Control n=152 n(%)	Total n(%)
next 2 weeks	7-day pp	27 (29·3%)	3 (7·3%)	30 (22·6%)
	3 mths prolonged	22 (23·9%)	3 (7·3%)	25 (18·8%)
next 30 days	7-day pp	36 (24·3%)	10 (22·2%)	46 (23·8%)
	3 mths prolonged	28 (18·9%)	9 (20·0%)	37 (19·2%)
next 6 months	7-day pp	45 (26·9%)	8 (14·0%)	53 (23·7%)
	3 mths prolonged	36 (21·6%)	8 (14·0%)	44 (19·6%)
not in next 6 months	7-day pp	6 (20·0%)	1 (11·1%)	7 (17·9%)
	3 mths prolonged	2 (6·7%)	1 (11·1%)	3 (7·7%)
